# Self-criticism of physicians, patient participation and risk competence

**DOI:** 10.3205/000210

**Published:** 2015-07-09

**Authors:** Michael Wolffsohn

**Affiliations:** 1 Munich, Germany

**Keywords:** self-criticism of physicians, patient participation, risk minimisation, social sciences, willingness to take risks, responsibility

## Abstract

Self-criticism of physicians and patient participation are the pillars of modern medical ethics and medical programmes. Patients expect risk minimisation from physicians, mostly without realising how much they could actively do themselves in this respect. But what about the willingness of German people to take risks, how high is it really at present? Direct empirical data are not available, but results from general empirical research show that people’s willingness to take risks is probably rather low. Post-heroic societies of welfare states are less likely to take risks than supposedly heroic ones. Therefore, the question whether it is responsible for medical experts to transfer even more responsibility to non-medical laypeople becomes increasingly important in a social context.

## Zusammenfassung

Mediziner-Selbstkritik und Patienten-Mitbestimmung sind die Grundpfeiler moderner Medizin-Ethik und -Programmatik. Von Ärzten erwarten Patienten eine Risikominimierung ohne meist zu realisieren, wieviel sie selbst aktiv beitragen könnten. Wie hoch ist aber derzeit die Risikobereitschaft der Bundesbürger wirklich? Direkte empirische Daten gibt es dazu nicht. Abgeleitet aus der allgemeinen historischen Empirie kann man aber annehmen, dass sie grundsätzlich sehr gering ist. Die postheroische Gesellschaft des Wohlfahrtstaates toleriert Risiken sicher weniger als die vermeintlich heroische. In diesem Zusammenhang bekommt die Frage, ob es zu verantworten ist, wenn die medizinisch Kompetenten noch stärker ihre Verantwortung auf die medizinisch Nicht-Kompetenten übertragen, eine gesellschaftliche Aktualität.

## Contemporary practices in society

Nowadays, everybody expects self-criticism of physicians and patient participation and rightly so because these practices correspond with the spirit of the age, the zeitgeist. People who fight against the zeitgeist face a moral dilemma because they become isolated. People who are isolated can hardly stay competitive in the market – if at all. Physicians refusing to submit to self-criticism or denying or even just reducing patient participation are placing themselves into the danger of economic isolation. Such isolation may affect both physicians in general practices and in hospitals as well as hospitals per se. In the end, medical progress would pay the bill because of the lack of financing. Thinking and acting according to the zeitgeist does not automatically equal material-oriented, discouraging or unprincipled opportunistic behaviour but is required in an economic sense. Self-criticism by physicians as well as patient participation based on the contemporary notions of zeitgeist are morally indefensible and even necessary. The absence of these practices would be inhuman because, without self-criticism of physicians and patient participation, patients would experience no or only little feeling of security. Patients consult physicians regarding concerns, care, prevention and aftercare as well as because of fear of disease and ultimately of death. Most people would prefer to live forever. Although physicians are not able to offer eternal life, they will try to prolong life and improve the quality of life. Patients expect from physicians at least the minimisation of risks but ignore that they are not only able but have to minimise risk themselves if they really want to live a life with less risk. This way, the risk competence of patients becomes a focus of medical debate and of reflecting on the possibilities and limitation of medicine.

## Self-criticism of physicians and patient participation

Consequently, current physician-patient-relations may be outlined as follows: Most patients no longer follow the instructions and prescriptions of physicians without question. Many, perhaps even most physicians grant their patients self-determination, co-determination and participation, not out of necessity but gladly and willingly. Furthermore, patient co-determination and co-decision are not only legal, that means required under and demanded by the prevailing law, but most physicians consider them legitimate, thus absolutely justified and necessary. These notions are not least documented in the texts provided in the context of this article. These texts also show the self-critical spirit despite claims to the contrary and in contrast to the ‘good old days’ (were these days really that good?).

Self-criticism of physicians and patient participation are the pillars of modern medical ethics and medical programmes. Physicians have long ceased to be the almighty omniscient ‘demigods in white’ who charge exorbitant fees. This development is more than welcome. To renounce these new virtues displayed by physicians would not only be anachronistic but would correspond to old times and thus contradict the current zeitgeist.

One question is often ignored in this context: Does self-criticism of physicians and patient participation include a higher risk for patients? Expert knowledge on the one hand, opinions on the other hand? The freedom of expression is not only always applicable but constitutionally protected. Yet, opinions and risk competence are not necessarily compatible. Does the medical-ethical twin pillar ultimately result in passing, unloading or shifting medical responsibility and medical risk from physicians to patients, from experts to laypeople? Does patient participation not completely overwhelm laypeople? Answering this question requires a radical critique of the self-criticism of physicians.

## Risk competence from different perspectives

Risk competence is the key word and the key issue of this multipart publication viewed from different perspectives. According to Ingrid Muehlhauser et al. [1], evidence-based health information must be science-based, independent, complete, true as well as understandable. But are these noble objectives not light years away from each other? Are experts generally able to explain their highly specialised knowledge to laypeople? Does this demand not have an element of squaring the circle?

Most of the time, it is the media which promise comprehensibility and keep the promise, not least by hardly acceptable simplifications. Is science becoming ignored in this process? The ingredients of baby food may be easily explained but not highly specialized therapy packages. Are they? Harald Schweim and Marcela Ullmann show the influence of the media on risk competence in the case of self-medication and make suggestions for improvements in this respect [2].

Ralf Stahlmann und Aniko Horvath state that understanding the toxic effects of substances necessitates sound physiological and biochemical knowledge [3]. There is no room for doubt regarding the accuracy of this statement. However, what about the realisation of patient participation as another important issue? Is such participation fiction? The current problems need greater awareness. Indeed, but how? Should managers, teachers, secretaries or construction workers study biochemistry alongside their work commitments? Are self-criticism and patient participation nothing more than a sham? Wouldn’t patients need two lives to be able to safe one life by additional studies? Here, if not before, medicine has its limits.

Michael Koller and Ulrich Hoffrage discuss social perspectives of risk perception and risk competence and claim that the paternalistic model is outdated and increasingly replaced by collaborative decision-making processes [4]. Isn’t such collaboration almost life-threatening for patients? At any rate, such collaboration is risky.

## Empirical data of social sciences

How compatible is collaborative decision-making with the risk averseness of ‘the’ Germans? What is the medical risk competence of ‘the’ Germans, and how high is their willingness to take risks? Historians with a social scientific background claim that ‘the’ Germans do not exist, similar to ‘the’ Americans, ‘the’ French, ‘the’ Jews and so forth. Generalised standardisations and serious analysis are mutually exclusive.

Meanwhile, however, empirically representative data are available, derived from more or less serious surveys on almost any topic in nearly every corner of the world, hence also in Germany. However, I am not aware of any surveys directly related to our topic, but the indirect empirical findings of the Institute for Demoscopy Allensbach give a clear picture. One of its employees, Petersen, summarised that‚ in some respects, there is a striking contrast between the fear of the (German; MW) population of different major life risks and the real dangers ([5], p. 380).

Next to its empirically representative status, historical science also involves other indicators, but not all of them meet the essential methodological requirements of empirical social sciences. We can only try to depict dominant social and cultural movements or zeitgeist trends. But how could such depictions become operationalised? This is the question. Often, the subjective factor, the subjective perception, the subjective wish of the analysing person is presented as a reality. However, such presentations are more illusion than reality, even when presented in a scholarly, educated and semi-referenced manner.

## Supposed willingness to take risks

Here, I will make the attempt to outline the real or supposed willingness of ‘the’ Germans to take medical risks. As far as I am aware, such an attempt can only be made in a historical context, without the employment of demographics, by using general facts to draw conclusions on individual aspects, that means from general historical developments to the specific willingness to take risks in a certain era. However, such an attempt will only show, if at all, the assumed willingness to take risks, both in a general and in a medical context. Sound facts are missing, and soft data have to be viewed with caution.

In a historical context, Germans living between the early 19^th^ century and 1945 appear to have been rather adventurous, even heroic, although the supposed heroic era of the Germans has been assumed to be the time before and particularly during the Anti-Napoleonic Wars of Liberation. However, these wars resulted in the liberation from Napoleon but not in freedom. But even in that time, first impressions did not correspond with impressions gained at a second glance. Most German men, particularly commoners, were not as enthusiastic about going to war as often proclaimed. If they went to war at all. Those who could shirked their military duties. It was mostly the poor who fought, thus little or not at all educated farmers and lower working class people (and their true enthusiasm may be questioned). But not everybody who was able to shirk his military duties refrained from expressing patriotic slogans and from sending others into the fight.

What do we conclude from this willingness to take risks? Germans living in those days were also people with a survival instinct that was much bigger than their death wish, people who – if they had the option – preferred to not take a risk in the case of danger of death. This pattern cannot only be found in German history but in the history of mankind. If people went to war – thus accepting life-threatening risks – it was mostly because of financial profit or because the obvious alternative to non-risk or non-fight was death.

Of course, not every risk constitutes a danger to life. However, historic-empirical research has shown that most people (although not everybody) do not like to even take innocuous risks. Although tranquillity is not the citizen’s first obligation, people obviously prefer to lead a quiet life. Unfortunately, due to the unavailability of individual proof, I have to derive such notions from the war and risk history of mankind including ‘the’ Germans. Obviously, we are faced with an anthropological constant, which may be applied to each case in a historical context throughout all epochs. Some people may disagree and refer to the legendary Prussian or German militarism: 1864 against Denmark, 1866 against Austria, 1870 to 1871 against France and the First and Second World Wars. Unfortunately (or thankfully), the German militarism of 1864 to 1918 has been debunked as a historical, empirical and economic legend by now. Without any doubt, the Second World War was caused by Hitler and his kind. However, from 1939 onwards, ‘the’ Germans went to war more anxiously than enthusiastically because many of them remembered the First World War and that war kills millions of people and is thus extremely dangerous. The death toll of the Second World War was 57 million lives. Both the voluntarily and the involuntarily heroic era came to an end in the German society as well as in other societies who had participated in the war. Ever since then, we live in a definitely post-heroic and anti-heroic era, that means, the willingness to take risks which, from an anthropological point of view, is rather low anyway is even lower than before the Second World War.

The decrease in the general willingness to take risks is also likely to include a decrease in the willingness to take medical risks. Taking a risk? Rather not, even a medical one, or only if there is no other alternative. I would like to mention one last contemporary counter-argument. In the context of the skiing accident of Michael Schumacher, the German newspaper ‘Welt am Sonntag’ printed the following headline on January 5^th^, 2014: ‘The popular sport of taking risks’. This perception and the well-researched and correct articles were nevertheless utterly wrong. Why was that? The authors committed a common methodological original sin because they compared the special (the absolute increase in the number of people taking part in dangerous sports) with the general, thus they compared part or parts with the whole. In a methodological context, it would be the same as if in resistance research ‘the’ Germans would be put on the same level as the few resistance heroes.

## Democracy in medicine

Of course, the risk of war is the highest of all possible risks of life and should be avoided at all costs, both for sensible and moral reasons. State – society – risk, this topic can be finally viewed from a different perspective: In the context of ‘welfare state’ or ‘social democratism’ within and outside of social democracy. Independent of the presence of social democrats in the government, the modern state is a welfare state, which reduces and minimises the daily risk of its citizens. In summary: Post-heroic societies of welfare states are even less keen on risk-taking in their daily life than assumedly heroic societies, who have often consisted of more braggarts than of true heroes. In post-heroic societies, hardly anybody wants to be a hero. I get the impression that this fact cannot be ignored, even in medical risk research.

From a contemporary point of view, self-criticism of physicians, patient participation, co-determination and self-determination are anything but new topics but belated reactions to and the acceptance of a process involving all sections of Western societies, thus also the society of (West) Germany, that started in the 1960s. For good or for bad, it is the victory of the 68-generation and the transfer of the request to ‘dare more democracy’ propagated by former Chancellor Willy Brandt in his first governmental declaration in October 1969. Democracy is transferred into medicine by politics and society at a late stage, maybe even too late? This is not original, but is it necessary? The articles published here shall help answering this question.

Is it responsible if medical experts transfer their responsibility to medical laypeople? This question cannot be simply rejected. This multipart publication can only be the starting point of further contemplation and discussion.

## Notes

### Competing interests

The author declares that he has no competing interests.
